# Escalation to Barbiturate-Induced Coma for Refractory Seizures after Liver Transplantation

**DOI:** 10.1155/2022/9311922

**Published:** 2022-01-10

**Authors:** Brittany Miles, Muhammad Mujtaba, Shehzad Merwat, Rupak Kulkarni, Jeffrey Fair, Michael Kueht

**Affiliations:** ^1^Department of Surgery, Division of Transplant Surgery, UTMB Galveston=University of Texas Medical Branch, Galveston, TX, USA; ^2^Department of Internal Medicine, Division of Transplant Medicine, UTMB Galveston=University of Texas Medical Branch, Galveston, TX, USA

## Abstract

Seizures after liver transplantation were previously thought to be a reliable harbinger of catastrophe, but more recent studies have found seizure activity to be relatively common, and most cases do not result in a poor outcome. Generalized seizures are the most common, and they typically occur de novo within the first two weeks after transplantation. The underlying cause for seizure activity in these patients may be complex, with potential etiologies including metabolic, infectious, cerebrovascular, and medication-induced causes. Identification of the underlying cause and the use of antiepileptic drugs (AEDs) is crucial for minimizing risk to the patient's neurologic and overall health. In this report, we present the case of a patient with refractory seizures unresponsive to conventional treatment, requiring prolonged barbiturate burst suppression with ventilator support. Seizure activity eventually ceased, and the patient made a full recovery.

## 1. Introduction

Seizures after liver transplantation were previously thought to be a reliable harbinger of catastrophe, but more recent studies have found seizure activity to be relatively common (up to 42% in some case series), and most cases do not result in a poor outcome [1, 2]. Generalized seizures are the most common, and they usually occur de novo within the first two weeks after transplantation [3]. The underlying causes for seizure activity in these patients may be complex, with potential etiologies including metabolic, infectious, cerebrovascular, and medication-induced origins [4]. Calcineurin inhibitors, which are commonly used in transplant patients for immunosuppression, are associated with Posterior Reversible Encephalopathy Syndrome (PRES), which is associated with neurologic symptoms in addition to characteristic MRI findings [5]. Identification of the underlying cause and the use of antiepileptic drugs (AEDs) is crucial for minimizing risk to the patient's neurologic and overall health. In this report, we present the case of a patient with refractory seizures unresponsive to conventional treatment, requiring prolonged barbiturate burst suppression with ventilator support. Seizure activity eventually ceased, and the patient made a full recovery.

## 2. Case Presentation

A 36-year-old male with a four-year history of alcohol abuse and an MELD score of 29 (See Appendix) presented for liver transplantation. At the time of evaluation, he had abstained from alcohol for more than 90 days, but his jaundice and coagulopathy persisted. He had a remote history of seizures but had been without any seizure events for more than 20 years and had not been taking antiepileptic medication during that time. Ten years prior, he underwent treatment for hydrocodone and buprenorphine use. He had been recently hospitalized for hepatic encephalopathy, and imaging showed signs of portal hypertension and chronic liver disease, including ascites, splenomegaly, and perigastric varices. Outpatient medications were as indicated in Table 1.

The donor of the blood-type-compatible allograft was a teenager who succumbed to head trauma. The only biochemical abnormality was a mild unconjugated hyperbilirubinemia (T. bili: 2.1, D. bili 0.3, and I. bili 1.8 mg/dL), and serologies were positive for anti-CMV IgG and anti-EBV IgG. The recipient underwent orthotopic liver transplantation utilizing a piggy-back cavoplasty technique with an operative time of 5 hours and 31 mins. Blood product requirements included two units of packed red blood cells, four of fresh frozen plasma, and two units of platelets. Vasopressors were used with a maximum norepinephrine dosage of 0.05 mcg/kg/min.

He was extubated within 12 hours of the operation and was taking clear liquids by postoperative day (POD) 2. Pain control initially required a significant amount of intravenous narcotic that was successfully weaned to oral medications by day five. The tacrolimus dosage was decreased on POD 5 due to tremor, and on POD 6, he was noted to have had four episodes of unresponsiveness, head deviation, and generalized shaking, each lasting approximately one minute. There was no incontinence or notable postictal state associated with these episodes, but they were determined to be focal seizures according to current guidelines [6, 7]. He was transferred to the ICU for monitoring and remained asymptomatic for the next 48 hours.

**Figure 1:**
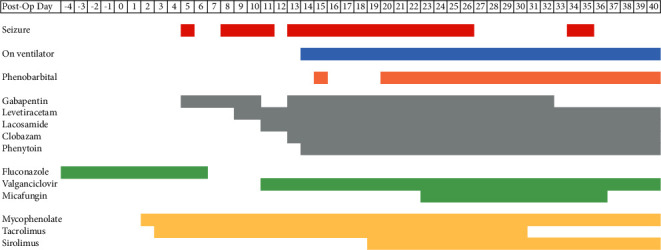
Timeline of seizures, medications, and ventilator use.

On POD 8, he again had focal seizures presenting as unresponsiveness with frothy oral secretions. Fosphenytoin was loaded with 20 mg/kg, and continuous electroencephalography (EEG) monitoring was begun. Over the next 24 hours, EEG revealed 8 focal seizures in the right frontotemporal and frontocentral regions that were not accompanied by convulsions. Levetiracetam was begun at 1000 mg BID. Epileptiform activity persisted on EEG, indicating progression to status epilepticus; levetiracetam was increased to 1500 mg BID, and lacosamide 200 mg q12 was added. On POD 13, seizure activity with left-sided localization remained present on EEG and gabapentin 600 mg TID and clobazam 5 mg BID were added for a total of four antiepileptic medications. Imaging of the head including Magnetic Resonance Imaging (MRI) and Computed Tomography (CT) was unrevealing aside from nonspecific scattered foci of T2/FLAIR signal abnormality in the bilateral cerebral white matter. Lumbar puncture revealed clear cerebrospinal fluid with normal glucose and protein with a single leukocyte per microliter. The use of propofol and phenobarbital for burst suppression (requiring endotracheal intubation) was initiated on POD 14, as shown in Figure 1.

The differential diagnosis list included posterior reversible encephalopathy syndrome (PRES), autoimmune/infectious causes, and benzodiazepine withdrawal.

## 3. Differential Diagnosis

### 3.1. Posterior Reversible Encephalopathy Syndrome (PRES)

Posterior Reversible Encephalopathy Syndrome (PRES) is a rare but very serious condition that can occur from the use of immunosuppressive agents used to prevent transplant rejection in both hematopoietic and solid organ transplant patients [5]. It typically presents as seizures, altered sensorium, visual abnormalities, or other neurologic changes affecting the occipital area of the brain, and imaging studies typically show changes consistent with vasogenic edema [8]. Although most often associated with calcineurin inhibitors, cases of PRES have also been reported with mycophenolate during the treatment of patients with systemic lupus erythematosis [9]. Our patient underwent multiple computed tomography (CT) head and magnetic resonance imaging (MRI) scans during his hospital course, and none showed any findings to support PRES as the underlying cause for his seizures.

### 3.2. Autoimmune/Infectious

Autoimmune encephalitis is another possible cause for refractory seizures, although it is most frequently described in bone marrow transplant patients and often presents with autoantibodies that can be found in patients' cerebrospinal fluid or sera [10, 11]. Such patients also tend to have characteristic findings on MRI, which may vary depending on the autoantibody involved [12]. Our patient did not exhibit signs of encephalitis on imaging studies. Additionally, his cerebrospinal fluid (CSF) analysis was analyzed twice and found to be negative for organisms and PMNs, in addition to having unremarkable autoimmune and serologic studies. The patient was also on multiple medications for immunosuppression due to the recent liver transplantation, which also contributed to this being an unlikely source of seizure activity.

### 3.3. Benzodiazepine Withdrawal

Withdrawal from benzodiazepines is another possibility for the development of seizure activity in this patient, although the greatest likelihood of seizure development occurs in patients taking high doses or short-acting benzodiazepines [13, 14]. This patient was taking alprazolam and chlordiazepoxide prior to liver transplantation, and it is possible that the discontinuation of those medications could have contributed to the development of seizure activity. However, benzodiazepine withdrawal is typically easily controlled by reintroduction of benzodiazepine [15]. In our patient's case, persistent seizure activity was seen despite the reintroduction of benzodiazepine therapy and required the dramatic intervention of burst suppression for seizure control.

On POD 13, the patient was receiving the 4-drug combination of gabapentin, lacosamide, clobazam, and levetiracetam, and frequent epileptiform activity was still present. Generalized convulsive status epilepticus (GSCE) can be treated with anesthetic-dose barbiturates, so the patient was started on phenobarbital and placed on a ventilator [16, 17]. He underwent tracheostomy after seven days of mechanical ventilation. To balance calcineurin inhibitor exposure with the needs of early posttransplant immunosuppression, sirolimus was added and tacrolimus was weaned off (see Figure 1). The patient's seizures ultimately ceased on POD 27, after 13 days of intensive barbiturate suppression. Gabapentin was discontinued on POD 32, and the final episodes of seizure activity occurred on POD 34 and 35. On postop day 40, the patient was weaned off the ventilator and no further seizure activity was noted. Although neurologic complications from prolonged barbiturate coma for refractory status epilepticus are common (33–78% of patients treated with this method), this patient primarily suffered sequelae of deconditioning including bilateral foot drop and left wrist weakness requiring splinting [18]. These improved with therapy, and he was walking prior to discharge. His cognitive function experienced a full recovery. He was discharged from the hospital on POD 72 with a plan to gradually wean the antiepileptics over the course of one year.

## 4. Discussion

Seizure activity after liver transplantation is a complex entity that can be a harbinger of catastrophe or can be easily treated. In this case, because the patient had preservation of liver function during these events, it was believed that the underlying cause of focal seizures progressing to bilateral convulsive status epilepticus was likely reversible. A thorough search for anatomic and infectious etiologies failed to reveal a cause, and so, a prolonged course of neurologic suppression was undertaken with the hope that if the other organ systems remained intact, the seizure threshold equilibrium would be reset. Although we may never know the underlying cause that led to refractory seizures in this patient, a possible theory is that the patient's right frontotemporal nidus of seizure activity was a previously dormant lesion that was unmasked by multifactorial causes including illness and seizure-threshold-lowering medications. Nevertheless, it is clear from this case that the use of burst suppression via barbiturate-induced coma was ultimately effective and resulted in the control of seizure activity after almost 2 weeks of intensive treatment.

## Figures and Tables

**Table 1:** Outpatient medications at the time of liver transplant.

Medication	Dose (mg)	Route	Frequency
Alprazolam	0.5	PO	TID
Dextroamphetamine-amphetamine	20	PO	Daily
Fluconazole	200	PO	Daily

## Abbreviations

AED: Antiepileptic drug
BID: Twice a day dosing
CMV: Cytomegalovirus
CSF: Cerebrospinal fluid
CT: Computed tomography
D. bili: Direct bilirubin
EBV: Epstein–Barr virus
EEG: Electroencephalography
FLAIR: Fluid-attenuated inversion recovery
GCSE: Generalized convulsive status epilepticus
I. bili: Indirect bilirubin
ICU: Intensive care unit
Kg: Kilogram
Mcg: Microgram
MELD: Model for end-stage liver disease
Min: Minute
MRI: Magnetic resonance imaging
POD: Postoperative day
PRES: Posterior reversible encephalopathy syndrome
T. bili: Total bilirubin.

## Appendix

MELD score of 29: Na: 143 mmol/L, INR: 3.2, Cr: 0.56 mg/dL, and T. Bili: 11.8 mg/dL.
